# Distribution and Effects of Estrogen Receptors in Prostate Cancer: Associated Molecular Mechanisms

**DOI:** 10.3389/fendo.2021.811578

**Published:** 2022-01-11

**Authors:** Adrián Ramírez-de-Arellano, Ana Laura Pereira-Suárez, Cecilia Rico-Fuentes, Edgar Iván López-Pulido, Julio César Villegas-Pineda, Erick Sierra-Diaz

**Affiliations:** ^1^ Instituto de Investigación en Ciencias Biomédicas, Centro Universitario de Ciencias de la Salud, Universidad de Guadalajara, Guadalajara, Mexico; ^2^ Departamento de Microbiología y Patología, Centro Universitario de Ciencias de la Salud, Universidad de Guadalajara, Guadalajara, Mexico; ^3^ Centro Universitario de los Altos, Universidad de Guadalajara, Tepatitlán de Morelos, Mexico; ^4^ Departamentos de Clínicas Quirúrgicas y Salud Pública, Centro Universitario de Ciencias de la Salud, Universidad de Guadalajara, Guadalajara, Mexico; ^5^ Departamento de Urología, Hospital de Especialidades Centro Médico Nacional de Occidente, Guadalajara, Mexico

**Keywords:** prostate cancer, estrogen receptor, castration - resistant prostate cancer, androgen deprivation therapies, GPER

## Abstract

Estrogens are hormones that have been extensively presented in many types of cancer such as breast, uterus, colorectal, prostate, and others, due to dynamically integrated signaling cascades that coordinate cellular growth, differentiation, and death which can be potentially new therapeutic targets. Despite the historical use of estrogens in the pathogenesis of prostate cancer (PCa), their biological effect is not well known, nor their role in carcinogenesis or the mechanisms used to carry their therapeutic effects of neoplastic in prostate transformation. The expression and regulation of the estrogen receptors (ERs) ERα, ERβ, and GPER stimulated by agonists and antagonists, and related to prostate cancer cells are herein reviewed. Subsequently, the structures of the ERs and their splice variants, the binding of ligands to ERs, and the effect on PCa are provided. Finally, we also assessed the contribution of molecular simulation which can help us to search and predict potential estrogenic activities.

## Introduction

Prostate cancer (PCa), is the second leading cancer-related disease among men and the fifth leading cause of death worldwide (1). This disease at the early stage can often be cured, however, those patients with metastasis need to undergo androgen deprivation therapy (ADT) (2), which progresses into a drug resistance stage, termed castration-resistant prostate cancer (CRPC). Although studies with an androgen receptor (AR) suggest that before ligand binding the receptor is located in the cytoplasm, that may in fact be a nucleus like downregulation of ERβ mRNA expression in hormone-refractory tumors, which provokes the acquisition of mesenchymal characteristics and aggressive behavior of the PCa cells (3) which could influence clinical symptomatic cancer and the initiation of PCa. Moreover, before nuclear translocation, ligand-bound steroid hormone receptors, both AR and ER can bind to homologous or heterologous ligands in the cytoplasm and exert biological effects (4).

ERs are activated by steroid hormones, such as estrogens, and mediate important physiological effects by binding to ERs (5). A specific ligand can exert an agonistic effect with an affinity depending upon the ER, implicated in non-receptor tyrosine kinase (Src) activation and phosphoinositol 3-kinase (PI3K) signaling pathway (6).

Therefore, it is important to improve this information so it can develop into potential tools for further investigation.

## Structural Diversity of Estrogen Receptors

ERα and ERβ are nuclear ERs, although both receptors interact with 17β- estradiol with high affinity, with the difference being in respect of the level of induction that may relate to the ligand (co-activation/repressors) (7). These receptors bind to specific sequences in gene promoters or through mechanisms that do not involve DNA binding (genomic and non-genomic effects) (8).

ERs are comprised of various domains and have several structural regions in common. The main functional domains are termed A/B, C, D, and E/F are present in both receptors’ full-length structures (4). The A/B region represents the amino-terminal domain (NTD) which is involved in transcriptional transactivation of gene. The C region corresponds to the DNA binding domain (DBD), contributing to the estrogen receptor dimerization. The D domain is a hinge region that connects the C and E domains, and it can bind to chaperone proteins allowing for the receptor-ligand complexes to translocate to the nucleus. The carboxy-terminal E/F region, also known as the ligand-binding domain (LBD), contains the estrogen binding area and binding sites for co-activators and co-repressors. Lastly, two additional regulators of estrogen activity, known as activation function (AF) domains, AF1 and AF2, are located within the NTD and DBD. The function of the former is hormone independent (AF1), and the latter requires the presence of hormone/steroid for transcriptional activation (AF2) (9).

**Figure 1:**
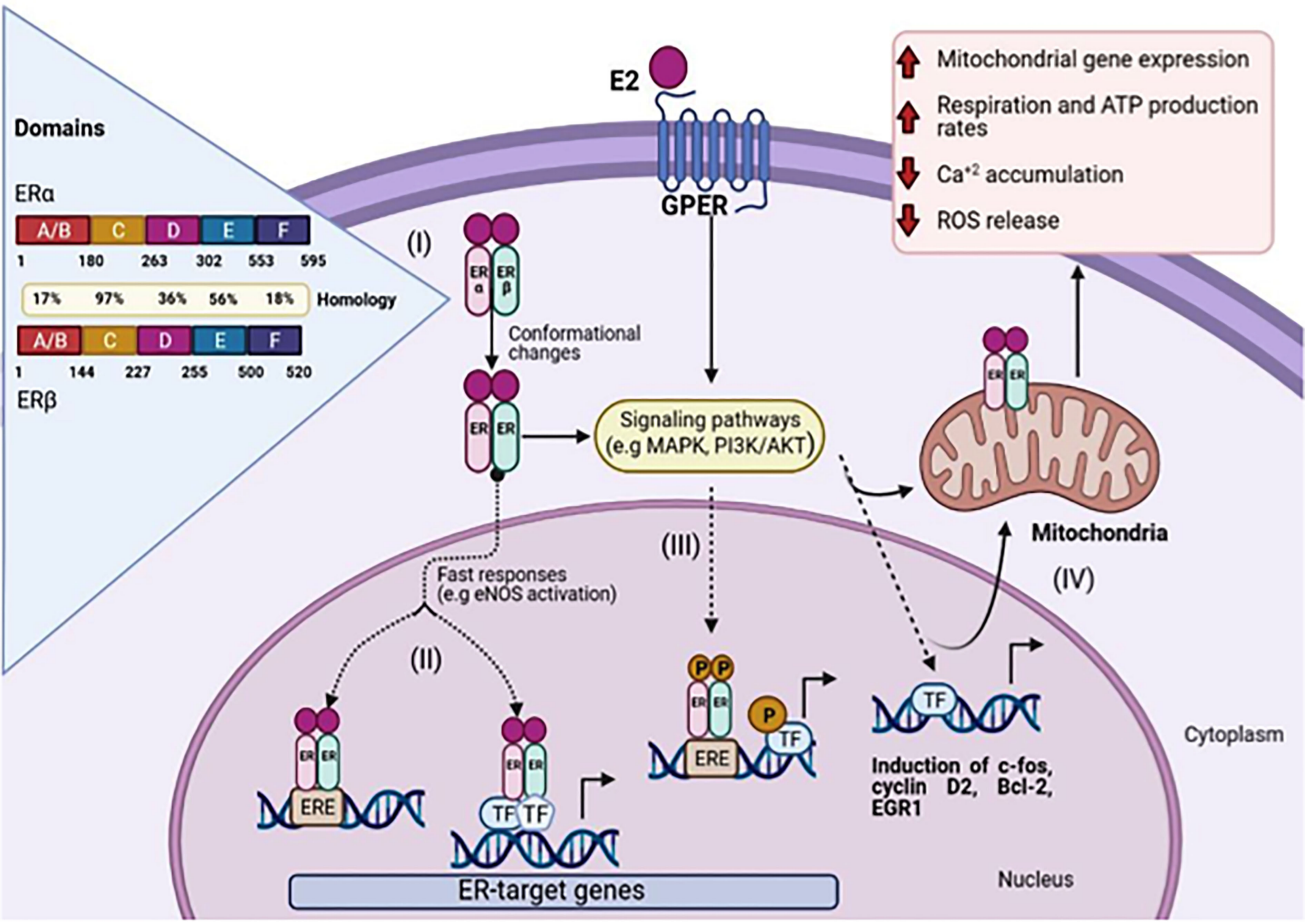
Schematic representation of 17β-Estradiol induced estrogen receptor-α, β, and GPER signaling. (I) The amino acid sequence position is shown for each domain and the homology between ERα and ERβ. Genomic pathway: (II) The E2/ER complex can bind to estrogen response elements (ERE) within the target gene promoter or regulate gene transcription by interacting with other transcription factors (TF), e.g. AP-1 and Sp1. (III) In addition, E2/ER complex activates signaling transduction pathways, leading to phosphorylation of ER or other bound transcription factors modulating gene expression. (IV) E2 initiated cellular and mitochondrial ER/GPER genomic and non-genomic actions modulate mitochondrial respiration, ATP production, and ROS formation. (Figure created with BioRender.com).

ERα and ERβ are isoforms of ER, which show high homology (more than 95% amino acid identity in the DBD domains and about 55% amino acid identity in the LBD) (4). (**Figure 1**, I).

The LBD is responsible for most of the functions activated by ligand binding, such as coregulator binding to AF2 (10) and dimerization interface, while DBD in ERs usually binds with high affinity to EREs and transactivates the gene expression in response to estrogen. The EREs sequencing performs an important regulatory role by dictating the binding affinity of ERs and modulating the recruitment of co-activators (4)(**Figure 1**, II).

Two mechanisms of action of the ligand-ER mediating the transcriptional regulation at ERE are reported. The binding of the ligand to the receptor triggers a variety of coregulators in a complex that alters chromatin and facilitates the transcriptional machinery. In this way, the Estrogen-ER complex acts as a transcriptional activator promoting gene expression (11), and another mechanism is by indirect genomic signaling of 17β estradiol, through protein-protein interactions with other classes of transcription factors at their respective response elements (12).

GPER is an estrogen receptor that was identified some decades ago. It belongs to the GPCR family (13). Unlike other family members, the GPER binding domain is present inside the plasma membranes (14) and the endoplasmic reticulum (15). After ligand binding to GPER, the activation stimulates cAMP production, calcium mobilization, and *c-Src*, which activates MMPs. It has also been demonstrated that these MMPs transactivate EGFR, which in turn activates MAPK and PI3K/AKT pathways that can induce non or genomic effects (16). (**Figure 1**, III), which causes diverse biological effects on the regulation of cell growth, migration, and programmed cell death in various tissues including the nervous, reproductive, digestive, and muscle systems (17).

GPER regulation of cell proliferation and apoptosis has also been linked with processes, namely, oxidative stress and Ca^+2^ homeostasis (**Figure 1**, IV). In the scenario of cell-fate maintenance of tissue homeostasis, the dual effects of GPER in the control of cell proliferation and apoptosis became evident, and consequently, depends on a panoply of factors that may include the type of tissue and a physiological context (18).

## Splice Variants in the Estrogen Receptor

Different ER variants have been reported. Recently, Qu et al. characterized circulating estrogen receptor mutants and isoforms in men from blood-derived RNA samples that were sequentially characterized from healthy and advanced PCa patients. The authors found six ERα mutations (E380Q, L536Q, Y537C, Y537S, Y537N, and D538G) in plasma from patients with advanced PCa, and six ERα and ERβ isoforms (ERα-66, ERα-36, ERβ1, ERβ2, ERβ4, and ERβ5) (19).

A novel splice variant of the human ERα, ERα30, was recently identified and cloned from clinical breast cancer tissue (20). The ERα-30 sequence lacks an LBD and a ligand-dependent transcriptional activation domain but retains the N-terminal transcriptional activation domain, the DBD, a partial hinge domain and possesses a unique 10-aminoacid domain. Nonetheless, different isoforms are generated not only by alternative splicing but also through post-transcriptional modifications (20).

The ERα-36 is a 36-kDa novel isoform of ERα that was identified and cloned by Wang et al. It is considered a new and important factor in understanding the pleiotropic effects of estrogen, even in organs without ERα-66 (21). Comparing human ERα-36 and ERα-66, the aforementioned lacks both transcriptional activation domains (AF-1 and AF-2) but retains the DBD and partial dimerization and the LBD, as in ERα-66 (22). Therefore, ERα36 expression is lower in tissues known to be predominantly regulated through ERα66 by the influence of non-genomic signaling (23).

ERβ may play a significant role in PCa affecting progression by expressing spliced variants. It has a full-length helix 11 and a helix 12 that assumes an agonist-directed position. In ERβ2, the C-terminus results in a disoriented helix 12 and it is marked in the coactivator binding cleft. ERβ4 and ERβ5 completely lack helix 12. Thus, ERβ behaves like a non-canonical type-I receptor. Its action may depend on differential amounts of ERβ1 homo and heterodimers formed upon stimulation by a specific ligand. Leung et al. suggest that all heterodimers have a higher transactivation activity than the ERβ1 homodimer, which serves as the “obligatory partner” of a functional dimeric complex, whereas ERβ2, ERβ4, and ERβ5 act as the “variable dimer partners” and serve as enhancers (24).

The cross-reactivity between different estrogenic ligand with ERα and ERβ isoforms in specific cellular contexts, and the fact that these receptors can recognize the same DNA-binding sites and interact with common co-regulators, seems to be a challenge, especially for those targeting hormone signaling pathways. In contrast, the splice variants are likely to be derived from non-malignant and malignant cells, especially as all six ER splice variants were detected in both healthy and ill patients (25), highlighting the importance of further research.

## Distribution of Estrogen Receptors and Effects on Prostate Cancer

The function of ERs depends upon their concentration and the presence of potential coregulatory proteins to ultimately repress or activate the expression of target genes (26).

ERα and ERβ have specific roles in PCa. It is known that ERα promotes proliferation, inflammation, and migration, whereas ERβ is considered antiproliferative and tumor-suppressive, and the loss of it has been associated with the progression to castration resistance (27) However, it has been considered that activation of ERβ increase non-phosphorylated β-catenin which promotes migration and invasion therefore it is currently consider as a controversy.

ERα 36 is localized in the membrane and contains the DNA-binding and dimerization domains from ERα by lacking both transcriptional activation domains (28), It is reported that high ERα mRNA and protein levels were detected in hormone-refractory and metastatic lesions with lymph node and bone metastatic samples (3). However, the exact role of ERα in prostate tumorigenesis is through MAPK/ERK, PI3K/AKT and β-catenin pathways, however the formation of metastatic lesions has not been investigated enough.

In benign prostate glands, ERβ1 was localized principally in the cytoplasm and nucleus of basal epithelial cells, but was also found in the perinuclear zone in luminal epithelial cells. Conversely, ERβ2 was localized predominantly in the cytoplasm of both basal and luminal epithelial cells. However, ERβ5 was localized almost exclusively in basal epithelial cells (27). The isoforms of ERβ in aggressive prostate increase cell migration and cell invasiveness. These findings established that ERβ2 and ERβ5 are strongly associated with PCa metastasis (29).

A recent study showed that the ERβ2 splice variant had oncogenic properties and was involved in osteolytic bone metastasis, in strong contrast to the tumor-suppressing effects of the other isoform ERβ1 (30). ERβ expression is located in dysplasia and was found to be associated with poor relapse-free survivals, when is located in cytoplasm and promotes aggressive behaviors of the cells with high-grade PCa (24, 31, 32).

A strong correlation between GPER and progression in the male reproductive system has been reported, which regulates testosterone and estrogen balance. Lower GPER expression was detected in benign prostatic tissues, and was mainly located in the epithelial basal layer, where this receptor could mediate estrogen action on normal cellular activity, and in neoplastic prostatic cells (33),. Therefore, benign prostate epithelial cells were noted to possess strong GPER immunoreactivity, indicating that expression of this receptor is inversely correlated to the degree of neoplastic cell differentiation (34). However, Chan et al. suggested GPER as a tumor suppressor by sustained activation of ERK1/2 and c-Jun/c-Fos-dependent, resulting in the arrest of PC3 growth at the G2 phase (35).

## ER-Ligand Assays Related to Prostate Cancer

ERα, ERβ, and GPER mediate the effects of estrogens in the normal prostate gland, a membrane-bound receptor, that is responsible for the rapid genomic and non-genomic actions of estrogens, which activate various protein-kinase cascades. During the progression of PCa, dynamic changes in both ERα and ERβ expression have been observed, which show a significant reduction of these receptors in tumor-associated stroma, compared to those of adjacent benign prostate. Additionally, ERα levels were higher in a higher-grade cancer compared to those at an early stage (36).

Conversely, the activation of ERβ induces anti-tumoral activity in PCa cells by repressing key oncogenes (*PI3K, p45Skp2, c-Myc*, and *cyclin E*, and the oncogenic *TMPRSS2-ERG* fusion), by increasing the expression of antiproliferative genes (*PTEN, FOXO3, KLF5, p21WAF1, CDKN1A*, and *p27Kip1*) as well as E-cadherin (37). Furthermore, ERβ signaling could be protective at an early stage of prostate carcinogenesis and it is shown that can switch during progression promoting migration, invasion and proliferation (38).

Interestingly, authors found the effect of agonist in ER which can modulate signaling pathways, such as ERα-selective agonist tri phenol (PPT) and ERβ-selective agonist diarypropionitrile (DPN) induce an increase on ERK1/2 but not AKT in the extranuclear region which would be important in order to develop new therapeutic strategies for the CRPC (39).

## GPER Ligands in Prostate Cancer

Chan et al. have previously reported that the stimulation of GPER by G-1, which is a specific agonist of GPER with no function on ERα and ERβ, impedes prostate cancer cell growth *in vitro* and *in vivo* assays (35). In a recently related study, Lau et al. reported that the binding of G-1 to GPER stimulates Gail proteins that sustains ERK1/2 activation but fails to activate adenylyl cyclase (AC) for cAMP production (40). Gail is expressed during PCa, and silences these proteins that inhibit cell growth of G-1. Furthermore, the stimulation of GPER by G-1 hampers the migration and invasion of PCa cells.

Chan et al. reported that activation of GPER with G-1 inhibits the growth of androgen-dependent and androgen-independent PCa cells *in vitro* and PC-3 xenografts *in vivo*, through sustained activation of ERK1/2, c-Jun/c-Fos-dependent up-regulation of cyclin-dependent kinase inhibitor 1 (p21), and induction of G2 cell-cycle arrest (35).

However, other signaling pathways resulted from the stimulation of GPER with other ligands have not yet been fully elucidated. In another research paper, they found, the relation between G-1 and androgen expression may impact G-1 *in vivo* studies, and as such, is yet to be made clear. Concerning LNCaP cell xenograft, the authors reported that during the androgen-sensitive (AS) versus castration-resistant (CR) phase, G-1 inhibited the growth of CR, contrarily to the AS tumors, and no toxicity of the host was noticed (41).

This inhibiting effect was associated with massive necrosis, neutrophil infiltration, up-regulation of a set of cell-mediated immune-response genes, and enhanced expression of GPER. The cell-based experiments revealed that GPER is repressed by androgen, whereas immunohistochemical studies, which found a larger proportion of human CRPC metastases than primary PCa, express high GPER levels (41).

Their results provided the first evidence that the GPER is an androgen-repressed target, and G-1 mediates the antitumor effect *via* neutrophil infiltration-associated necrosis in CRPC. Conversely, the lack of acetyl group in G-15 compared to G-1 prevents biological responses mediated by GPER in cancer cells and *in vivo* like epithelial uterine cell proliferation and anti-depressive effects in mice, contrarily to G-1 and estrogens. In the previous reference, the authors suggest that the antagonist of GPER may display similar selectivity against uterine and neurological responses initiated by estrogen.

## Associated Mechanisms Between ER and GPER in Prostate Cancer

**Figure 2:**
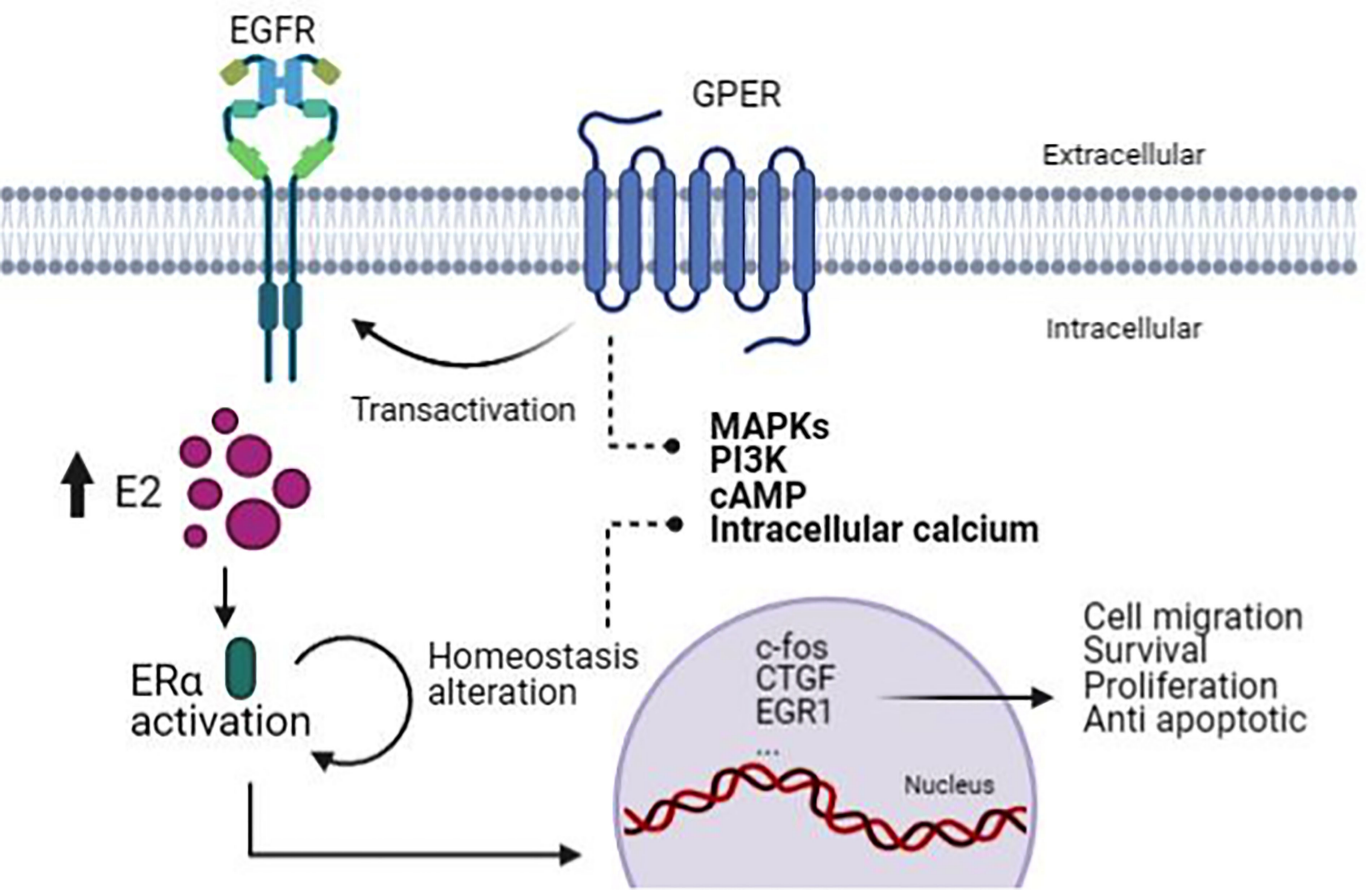
The mechanism associated with ER and GPER in PCa. Due to the increasing activity of ERα, this brings about homeostasis alteration that GPER transactivates EGFR, causing activation of pathways involved in cell migration, survival, proliferation, and anti-apoptotic process. (Figure created with BioRender.com).

Estrogens are critical hormones that regulate the development of hormone-sensitive tumors. It is known that any compound that up-regulates aromatase levels will not only elevate intracellular E2, but will also increase the activation of the mainly proliferative, classic ERα to induce adipogenesis and growth disorders in estrogen-sensitive tissues, as well as GPER that alters important intracellular signaling sequences that promote mitogenic growth and endothelial damage (42). Unfortunately, the physical effects of illness and inflammation activate aberrant signal transduction and anti-apoptotic processes by altering serine/threonine phosphorylation cascades of multiple tyrosine kinase receptors that act through MEKK/ERK and PI3K/Akt/mTOR/NF-kB pathways (43) (**Figure 2**).

Therefore, differentiating functions of splice variants for estrogen receptors will further clarify our understanding of their roles in PCa progression. While the role of ERβ in PCa is well studied, there are relatively few studies on the functional role of ERα in prostate tumorigenesis (44).

Through ERα-36 that induces the initiation of non-genomic signaling pathways to activate MAPK/ERK and PI3K/AKT pathways, this regulates c-Myc protein expression, thereby contributing to the metastatic potential of cancer. The interaction of ERα-36 with E2 causes Src activation inducing downstream cascades: MEK activation, phosphorylation of ERK and paxillin (PXN), which induces third messenger expression, cyclin D1 (45) and protein kinase C (PKC) through ERK1/2 leading to an increase in cyclin D1/cyclin-dependent kinase 4 which control cell cycle progression, proliferation, metastasis, and survival (46)

Moreover, several studies suggest that ERα-36 could act as a negative dominant regulator of estrogen genomic signaling promoted by ERα-66 and ERβ. However, there is a positive feedback mechanism between ERα-66 and ERα-36. ERα-66 appears to be able to suppress ERα-36 activity, and the loss of ERα -66 expression increases ERα -36, which represents one of the resistance mechanisms to antiestrogen therapy (46). To our knowledge, the signaling related to ERα-66 in PCa needs to be researched.

ERβ isoforms in PCa are known to down-regulate the progression of this disease and the expression of androgens receptors (AR) (47), as seen in ERβ1, whose depletion increases AR. In addition, ERβ1 has been shown to induce apoptosis in PCa cell lines by activating the FOXO3 and PUMA pathway (36) and inhibit EMT by upregulating prolyl hydroxylase domain 2 (PHD2/EGLN1), and subsequently decreasing hypoxia levels (24). Alternatively, ERβ2 is expressed in metastatic prostate cancer and its location in the nucleus correlates with decreased survival (29), increased invasiveness, cell proliferation, expression of *Twist 1* and *c-Myc* in PC3 and 22Rv1 cells, thus indicating possible oncogenic roles of ERβ2 in PCa (30).

It is shown that ERβ2 interacts and stabilizes HIF-1α protein in normoxia by inducing a hypoxic gene expression signature. HIF-1α stimulates metastasis by increasing Twist1 expression and increasing vascularization by activating VEGF expression (48). These findings suggest that ERβ2 is mediated by HIF-1α, and these interactions may be a strategy for treating PCa.

The increased activity of ERα and ERβ can alter the homeostatic balance of the GPER transmembrane regulatory cascade, and in turn, these pathways increase growth, stimulate anti-apoptotic processes, activate mitogenic change, and metaplastic alteration, and well as neoplastic activity in estrogen-sensitive tissues (6).

For instance, an interesting co-dependence between GPER and ERα toward the stimulation of gene expression and cell proliferation has been revealed, and it includes the up-regulation of *c-Fos*, *CTGF*,*Egr1* (17) and other genes involved in important biological responses.

## Molecular Simulations of Estrogen Receptors

Molecular simulation is a handy and powerful tool. Nowadays, theoretical calculations on this topic can provide some insights into the binding of a ligand to a receptor and help develop novel specific drugs, which is currently one of the greatest challenges of the pharmaceutical industry. The binding of a ligand to the ERs occurs in the LBD, which presents a canonical antiparallel a-helical triple sandwich topology, similar to other NRs. The interactions of ERα and ERβ with ligands are commonly the targets in molecular simulation.

Lin et al. explores a prostate-associated gene, an intrinsically disordered protein implicated in PCa. They performed molecular dynamics simulations to understand the interactions underlying this structural transition, which show that electrostatic interaction drives the transient formation of an N-terminal loop, that causes a dramatic change in size upon hyperphosphorylation. This leads to constructing a mechanism-based mathematical model that allows capturing the interaction of different phosphorylated forms of PAGE4 with AP-1 and its downstream target, the androgen receptor, a key therapeutic target in PCa (49). Their model predicts intracellular oscillatory dynamics indicating phenotypic heterogeneity in an isogenic cell population, in order to gain insights into its functional implication that may modulate hormone expression in PCa cell though future therapy interactions.

## Estrogens and Prostate Cancer Patients

Prior to ADT, prostate cancer was managed using estrogen treatment. The secondary cardiovascular effects were a serious concern for therapy, since its use was limited to prostate cancer patients (50). Regarding the clinical field and novel research, a type of androgen-dependent tumor remains as a paradigm for PCa. Although the role of estrogen on PCa development is important, scientific information is scarce. The association between plasma estrogens remains unclear and sometimes contradictory, since circulating testosterone in plasma decreases with age. Increasing the ratio of estradiol testosterone leads to aromatase activity and higher conversion of testosterone to estradiol (51, 52). Malignant and non-malignant prostate cells are provided with key enzymes of steroid metabolism (17ß-hydroxysteroid dehydrogenases, 5ß-reductase, and aromatase). Metabolic activity of these compounds may in the end lead to a differential prostate accumulation of steroid by-products with no clear behavior (51).

Nowadays, the clinical field regarding PCa management is broader. Around 15 years ago, progression to androgen deprivation management in metastatic castration-resistant prostate cancer (mCRPC) cases, were managed based on hormonal manipulation with antiandrogens such as nilutamide and bicalutamide (53). New chemical compounds have been approved for high risk mCRPC, including enzalutamide and abiraterone, which are associated with longer disease control and survival if combined with androgen deprivation therapy (54). The targeted androgen receptor inhibitor enzalutamide blocks the androgen receptor binding to DNA, and androgen receptor translocation to the cell nucleus. Abiraterone is a selective inhibitor of CYP17. Its effect on CYP17 results in an increased level of mineralocorticoids, due to high levels of the adrenocorticotropic hormone (53). Inhibition of CYP17 blocks androgen synthesis by producing tumor responses in prostate cancer patients who no longer respond to standard hormonal therapies (55).

In 2021, Jurečeková et al. revived scientific focus on estrogen activity in PCa, subject to the possibility that an intraprostatic estrogen milieu might have a role that is more important than only circulating estrogen levels. They determined the ESR2 expression levels in malignant prostate tissues and analyzed a possible association with *ERS2* gene polymorphisms (52). The case control study, which included 510 prostate cancer patients, concluded that the polymorphism rs3020449 in the *ESR2* gene should be considered as a risk factor for prostate cancer in Slovak males. This polymorphism was associated with high grade carcinomas (Gleason score >7) and stages pT3/pT4.

As previously mentioned, current cancer research is focused on androgen receptor signaling. Evidence of estrogen and estrogen receptors in prostate cancer pathology is not conclusive, nor are there any clear results, due to a lack of information. A new compound for prostate cancer treatment such as abiraterone acetate, which induces a wide effect on several hormones and its receptors, could be the first step towards estrogen receptors interest for future research.

## Discussion

The present review highlights the recent research advances in prostatic cancer and provides an overview for further research work related to this topic. It has been recognized for many years that ER is a predictor of hormone responsiveness. Furthermore, there is ER expression in positive cancer that does not respond and generate resistance, and unfortunately, the consequences of hormonal therapy is that many patients who do respond will eventually relapse in consequence of the development of resistance (3). This is unlikely to be due to major losses of Erα, since the immunohistochemical analysis of paired biopsies taken before tamoxifen treatment and following relapse showed a loss in only about 20% of the cases (56).

Alternatively, ERβ led investigators to hypothesize that this might be involved in phenotypic resistance, and was associated with poor prognosis and an elevated expression of ERβ (57). Also, the responses mediated by GPER have a critical interaction in PCa, since it plays an important role in tumor development and metastasis by activating signaling pathways involved in exacerbated proliferation, resistance to apoptosis, stimulated migration and invasion, induction of angiogenesis, and metabolic reprogramming (24).

The screening of various compounds by molecular simulation is beneficial to finding promising selective agonists or antagonists, and significantly contributes to successful drug design.

It is widely known that estrogen is associated with carcinogenesis through specific mechanisms, and multiple variants of ER are involved in pathophysiology and signaling pathways in PCa. GPER is expressed both in the normal gland and PCa. Overall, a deeper knowledge of the roles of ERα or ERβ and their interaction with GPER is required in order to apply future therapies to PCa. Molecular simulations will aid in deciding which receptor subtype should best be targeted for the management or cure of this pathological disorder.

However, *in vitro* and *in vivo* bioassays are undoubtedly indispensable. It should be mentioned that the activity elicited by ER antagonists, though ERα and GPER as stated above, could represent a therapeutic concern with regard to the pharmacological manipulation of cancer cells, through inhibiting all types of estrogen receptors, however, in PCa, it has not yet been fully elucidated

## Author Contributions

All authors participated directly in the manuscript. AR-d-A, AP-S, and ES: Conceptualization, project administration and writing-original draft preparation. ES: Editing. CR-F, EL-P, and JV-P: Investigation and writing-review. CR-F: Digital art (BioRender^®^). All authors have read and agreed to the published version of the manuscript.

## Funding

This work was supported by Jalisco Scientific Development Fund (FODECIJAL) to Attend State Problems 2019 (Project #8164).

## Conflict of Interest

The authors declare that the research was conducted in the absence of any commercial or financial relationships that could be construed as a potential conflict of interest.

## Publisher’s Note

All claims expressed in this article are solely those of the authors and do not necessarily represent those of their affiliated organizations, or those of the publisher, the editors and the reviewers. Any product that may be evaluated in this article, or claim that may be made by its manufacturer, is not guaranteed or endorsed by the publisher.
